# Accelerating myelin defect detection in neurodegenerative disorders: a human-in-the-loop deep learning approach with birefringence microscopy

**DOI:** 10.1117/1.NPh.12.4.045007

**Published:** 2025-11-04

**Authors:** Anna Novoseltseva, Arjun Chandra, Alexander J. Gray, Shuying Li, Mikayla Bradsby, Irving J. Bigio

**Affiliations:** a Boston University, Department of Biomedical Engineering, Boston, Massachusetts, United States; b Boston University, Department of Computer Science, Boston, Massachusetts, United States; c Boston University, Department of Electrical and Computer Engineering, Boston, Massachusetts, United States; d Boston University, Department of Physics, Boston, Massachusetts, United States

**Keywords:** myelin, birefringence microscopy, Alzheimer’s disease, chronic traumatic encephalopathy, deep learning, YOLOv8, human-in-the-loop

## Abstract

**Significance:**

Myelin degradation is a critical yet understudied pathological feature in neurodegenerative disorders. Manual detection of myelin defects in volumetric microscopy images is prohibitively time-consuming, limiting large-scale studies. There is a need for rapid, accurate, and scalable defect-detection methods to accelerate advances in the field.

**Aim:**

We aim to develop and evaluate a human-in-the-loop deep learning approach to accelerate myelin defect detection.

**Approach:**

We imaged brain tissue samples from the dorsolateral prefrontal cortex from 15 subjects (i.e., five controls, five Alzheimer’s disease, and five chronic traumatic encephalopathy) using RGB circular crossed-polarized birefringence microscopy. We created a dataset of 5600 manually annotated myelin defects and trained a YOLOv8-based defect detection model with iterative expert verification.

**Results:**

Our approach achieved 0.85 mAP@50 and reduced analysis time from 8 h to 33 min per $1\;{\mathrm{mm}}^{2}$ of tissue while maintaining high accuracy for disease comparison studies. The method can process complete 3D volumetric images up to 300 GB, enabling comprehensive assessment across large tissue volumes.

**Conclusions:**

This approach effectively streamlines myelin defect detection and can enable the scale up of myelin degradation studies in neurodegenerative disorders.

## 1 Introduction

Myelin degradation represents a critical pathological process underlying numerous neurological conditions, from inflammatory diseases such as multiple sclerosis to neurodegenerative disorders such as Alzheimer’s disease (AD) and chronic traumatic encephalopathy (CTE).^1^^–^^4^ Growing evidence demonstrates that myelin dysfunction is not merely a consequence but may play an important implicatory role in neurodegeneration, with recent studies showing that myelin breakdown directly promotes amyloid-β deposition in AD models^5^ and contributes to cognitive decline across neurodegenerative conditions.^6^^,^^7^ Myelin degradation occurs through multiple pathological mechanisms, including inflammatory demyelination,^2^ metabolic dysfunction that compromises oligodendrocyte viability,^3^^,^^8^ and lipid alterations that disrupt myelin integrity.^5^^,^^7^ These processes manifest as characteristic morphological alterations, including myelin breaks, delaminations, blebbings, swellings, and irregularities, which directly impair saltatory conduction and contribute to functional decline.^8^^,^^9^ This emerging understanding creates an urgent need for efficient and accurate methods to quantify myelin pathology at the microscopic scale but over significant volumes of tissue.

Current methodological limitations create significant hindrances due to the high cost and laborious nature of current approaches in myelin imaging. Although magnetic resonance imaging (MRI) techniques provide valuable clinical insights, they lack resolution and sensitivity for detecting the subtle microstructural alterations characteristic of early disease stages.^10^^,^^11^ Electron microscopy represents the gold standard for detailed ultrastructure analysis but requires expensive specialized equipment and extensive sample preparation^12^^,^^13^ and is not practical for examining large tissue volumes.

Birefringence microscopy is recently emerging as a wide-field, unlabeled imaging modality that can resolve individual myelin defects and is more scalable than other techniques.^14^ Most critically, however, based on our previous validation study,^12^ manual analysis of myelin defects in microscopy images is prohibitively time-consuming, requiring ∼8  h per $1\;{\mathrm{mm}}^{2}$ of tissue section,^9^ which severely limits the scale and statistical power of pathological studies.

Recent technological advances have created an unprecedented opportunity to address this bottleneck through the convergence of accessible birefringence microscopy and advanced computer vision. Circular crossed-polarized birefringence microscopy (CCP-BRM) takes advantage of the strong birefringence of myelin as a contrast mechanism and offers transformative capabilities by providing single-axon resolution for myelin visualization while preserving tissue architecture.^15^^,^^16^ Critically, this technique can be deployed on standard wide-field microscopes with the addition of simple circular polarizers, making high-resolution myelin analysis accessible to any laboratory.^15^ However, CCP-BRM, by itself, does not provide quantitative analysis of fiber orientations, and existing quantitative methods require at least three sequential image acquisitions at different angles of linear polarization illumination,^14^ limiting real-time visualization. A novel implementation is desired to provide real-time color-encoded fiber orientation visualization.

Following principles established by Higgins^17^ for mineral analysis, the addition of a color camera and white light source to the CCP-BRM microscope allows real-time fiber orientation visualization. This is affected by the wavelength-dependent polarization effects arising from the dispersion of birefringence of the quarter-wave plate (part of the circular polarizer illumination) as well as the myelin fibers. Given that quarter-wave plates are optimized for a specific wavelength, they provide different degrees of ellipticity for other wavelengths in a broader wavelength light spectrum.^18^ When this wavelength-dependent illumination interacts with birefringent structures, it introduces additional retardance described by

$$\delta =\frac{2\pi d(n_{e}-n_{o})}{\lambda },$$ (1)

where d is fiber thickness, $\left(n_{e}-n_{o}\right)$ is the birefringence index difference—the value of each is wavelength dependent—and λ is the wavelength.^19^ Each wavelength is characterized by a distinct polarization state that interacts differently with the analyzing circular polarizer.

The color-encoding mechanism occurs through multiple wavelength-dependent effects: For a quarter-wave plate optimized at $\lambda_{0}$, shorter wavelengths ($\lambda <\lambda_{0}$) experiences an increase in retardance, whereas longer wavelengths ($\lambda >\lambda_{0}$) experience a decrease in retardance, creating wavelength-specific elliptical polarization states in the illumination. As this spectrally encoded illumination passes through myelin fibers, the basic retardance relationship [Eq. (1)] ensures that different wavelengths accumulate different phase delays, with blue light (shorter λ) experiencing greater retardance than red light (longer λ) for the same optical path. The orientation dependence arises because fibers at different angles relative to the polarization axes contribute varying amounts of retardance, creating the dynamic color transformations that encode fiber orientation without the extinction positions characteristic of crossed linear polarizers.

Simultaneously, advances in computer vision—particularly You Only Look Once (YOLO)-based object detection models—have demonstrated remarkable success in medical imaging applications, including brain tumor detection in MRI, breast cancer identification in mammograms, COVID-19 detection in chest radiographs, and cellular analysis in microscopy images.^20^ Recent studies show YOLO network architectures achieving high accuracy for cellular detection in microscopic images, including blood cell identification, fluorescence microscopy analysis, and recognition of pathological features.^21^^–^^23^

Recently published work used YOLO for myelin defect identification in the corpus callosum of a nonhuman primate model.^24^ That brain region is characterized by highly parallel white-matter fiber organization, and the study established proof-of-concept for object detection approaches in birefringence microscopy^24^ of myelin defects and lipid vesicles derived from myelin debris. However, extending that approach to the complex environment of human cortical pathology is challenged by the diversity of fiber orientations, post-mortem tissue deterioration, and varied pathological patterns.

The integration of birefringence microscopy and deep-learning-based object detection through human-in-the-loop methodologies represents a promising paradigm for addressing complex morphological analysis tasks. Although traditional fully automated approaches may sacrifice accuracy for speed, human-in-the-loop frameworks preserve expert knowledge while dramatically improving efficiency.^25^^,^^26^ This approach has shown promise, especially in medical imaging applications, where expert validation remains essential.^27^^,^^28^

We report the development of a human-in-the-loop deep learning approach specifically optimized for myelin defect detection in complex human cortical tissue, based on imaging with accessible birefringence microscopy. Our microscope combines white-light illumination and a color (RGB) camera with green-band crossed-circular polarizers for illumination and detection to generate RGB-encoded circular crossed-polarized birefringence microscopy (RGB CCP-BRM) for real-time fiber visualization. This imaging method is combined with YOLOv8 object detection and iterative expert verification, demonstrating that pathological analysis can be achieved with a 14-fold time reduction while preserving the accuracy required for comparative disease studies. This advance will facilitate comprehensive investigations of myelin–pathology relationships that were previously impossible due to throughput limitations.

## 2 Materials and Methods

Our integrated approach combines birefringence microscopy with deep learning automation to create a practical solution for high-throughput myelin analysis. The methodology encompasses four core components: (1) RGB and grayscale CCP-BRM imaging, (2) human-in-the-loop annotation workflows, (3) deep learning model training with domain adaptation, and (4) validation against expert consensus.

### 2.1 Study Design and Workflow Overview

The human-in-the-loop workflow integrates manual annotation, deep-learning-based pseudo-labeling, and expert verification to achieve myelin defect detection with dramatically improved efficiency. RGB CCP-BRM serves as the primary imaging approach with color-encoded fiber orientation, whereas grayscale adaptation enables validation against established expert consensus datasets.

**Fig. 1:**
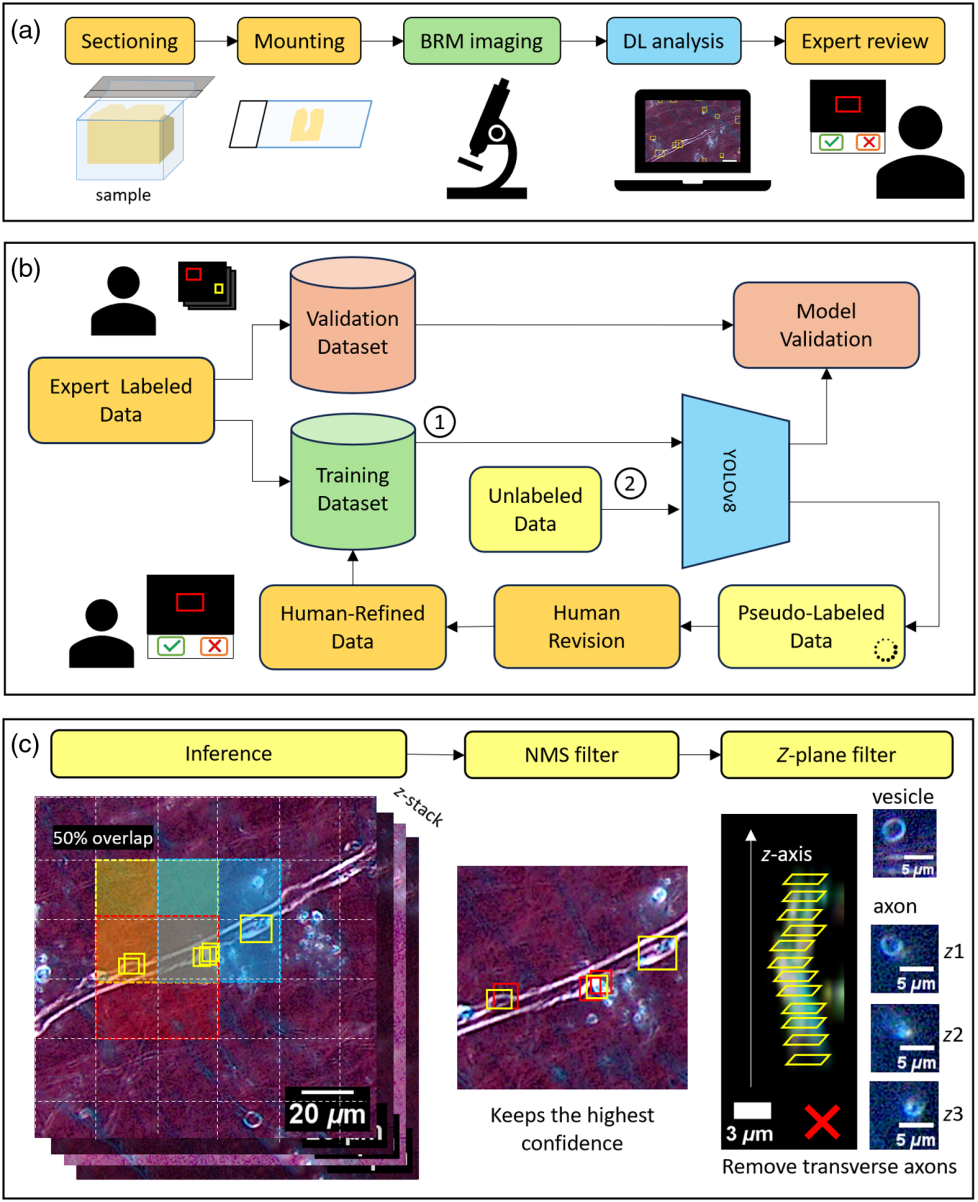
Human-in-the-loop workflow for automated myelin defect detection using birefringence microscopy. (a) Complete pipeline overview from tissue preparation to final analysis. Brain tissue samples undergo sectioning, wet-mounting with index-matching, circular crossed polarized birefringence microscopy (CCP-BRM) imaging, deep learning (DL) analysis using YOLOv8, and expert verification with accept/reject decisions. (b) Iterative training workflow demonstrating the human-in-the-loop approach. Expert-labeled data initializes model training (1), followed by pseudo-labeling of unlabeled data (2). Human revision of model predictions generates human-refined data that feeds back into the training dataset, creating a continuous improvement cycle. The validation dataset remains separate for unbiased performance evaluation. (c) Inference and post-processing pipeline for comprehensive tissue analysis. Sliding window inference with 50% overlap ensures complete tissue coverage (left). Nonmaximum suppression (NMS) filtering retains the highest confidence detections while removing redundant predictions (center). Z-plane filtering eliminates false positives from transverse axons appearing as circular structures across multiple focal planes (right). Images on the right demonstrate the visual appearance of the lipid vesicle and transverse axon in three different z-planes, noted as z1, z2, and z3. Yellow boxes indicate detected defects.

Figure 1 shows the complete pipeline from tissue preparation to final analysis, highlighting the iterative human-in-the-loop training process and comprehensive inference pipeline.

### 2.2 Human Brain Tissue Samples

Human brain tissue samples were obtained from the Boston University Alzheimer’s Disease Research Center and UNITE brain banks following institutional protocols. The cohort included 15 subjects comprising five normal controls, five neuropathologically confirmed late-stage Alzheimer’s disease (AD) cases, and five chronic traumatic encephalopathy (CTE) cases. The demographics information is provided in Table 1. All tissue samples originated from the dorsolateral prefrontal cortex (Brodmann areas 9 and 46) and were ∼2×2×0.5  cm in size.

**Table 1:** Subject demographics.

Group	Sample #	Sex	Age, y	PMI, h	Braak stage	CTE stage
Control^a^	1	F	61	4		
	2	M	69	13.5		
	3	M	67	13.5		
	4	M	59	13.92		
	5	M	88	17	IV	
CTE	6	M	81	15.75		III
	7	M	89	17.5		III
	8	M	78	7		IV
	9	M	75	16.5		IV
	10	M	86	18.75		III
AD	11	F	84	6.5	VI	
	12	M	76	3	VI	
	13	M	86	6.25	VI	
	14	F	83	10.5	VI	
	15	M	65	17.75	VI	

PMI, post-mortem interval.

a Controls are defined by the absence of NFTs and Aβ pathology in the dorsolateral prefrontal cortex; that and the absence of neurodegenerative disorder diagnosis are the main selection criteria.

The samples were cut into 30-μm-thick sections (∼2×2  cm area) using a vibratome, wet-mounted, and index-matched in 85% glycerol (to minimize scattering), following established protocols.^16^ These tissue samples and the associated expert consensus annotations were previously utilized in our validation study of manual CCP-BRM analysis,^9^ enabling direct comparison between manual and automated approaches on identical datasets.

### 2.3 Imaging System and Parameters

**Fig. 2:**
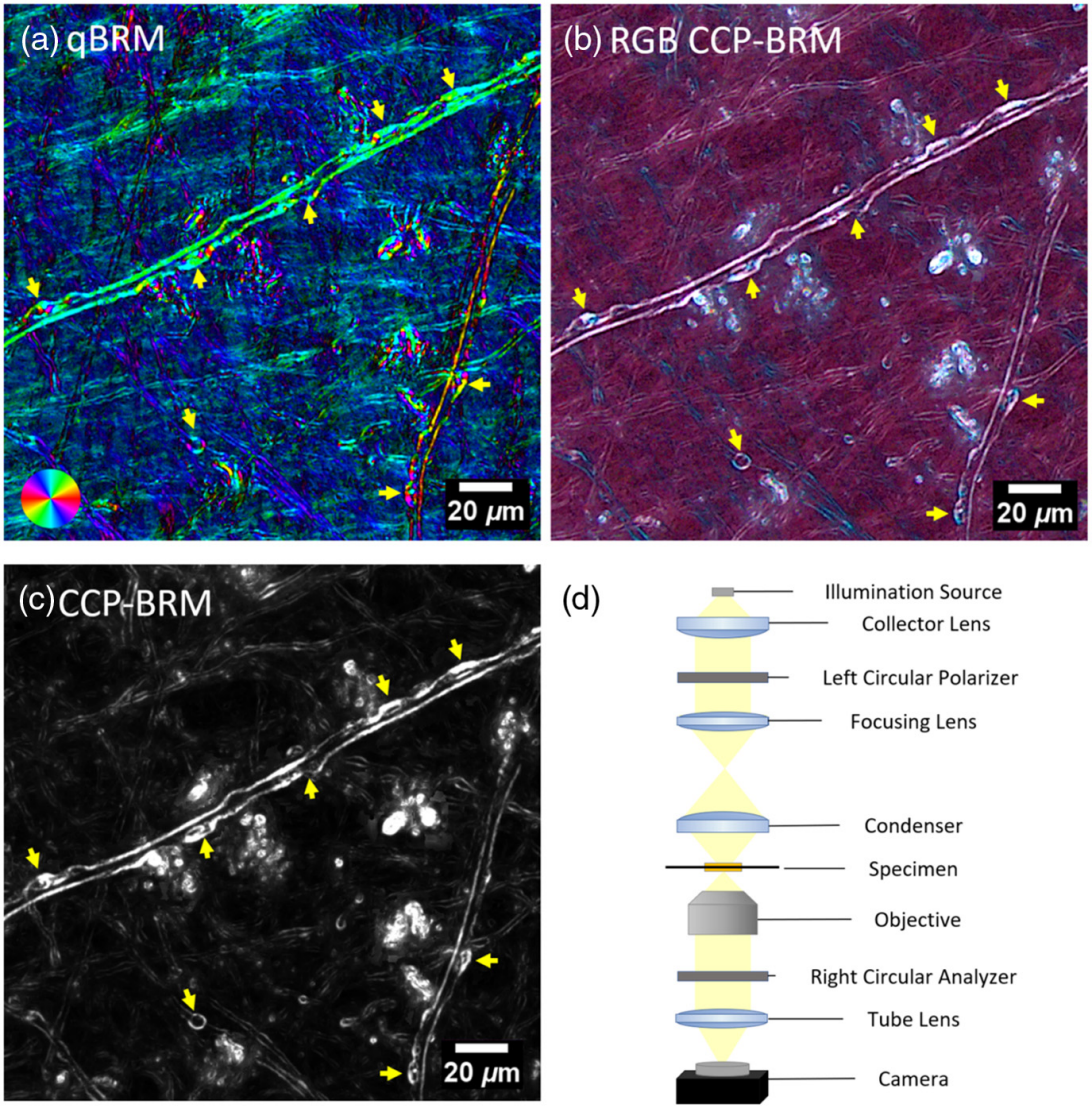
Comparative imaging modalities demonstrate enhanced myelin defect visualization with RGB CCP-BRM. (a) Traditional qBRM image of AD patient tissue showing myelin architecture after computational post-processing (color wheel indicates optic axis orientation). (b) RGB CCP-BRM image of the same region of interest, demonstrating enhanced defect visibility with real-time color-encoded fiber orientation information. (c) Grayscale CCP-BRM image of the same ROI showing improved contrast compared to qBRM. (d) Schematic diagram of RGB CCP-BRM imaging system: white light source illuminates tissue through the left circular polarizer, birefringent tissue structures modulate polarization states, and the right circular analyzer analyzes the signal before detection by the color camera. CCP-BRM approaches (b), (c) provide sharper structural defect details compared with qBRM (a) due to differences in optical configurations, whereas RGB CCP-BRM offers additional advantages through wavelength-dependent polarization effects that encode fiber orientation as color variations, making structural disruptions more apparent to both human annotators and automated detection algorithms. Scale bars: 20  μm.

CCP-BRM imaging was performed using a commercial slide-scanner microscope (Olympus VS-120) modified with circular polarizers.^9^ The RGB CCP-BRM implementation utilized “white” light illumination (XCite 120LED light engine) and a color camera (AVT Pike F-505C), providing real-time fiber orientation encoding with operational advantages over traditional quantitative birefringence microscopy (qBRM)^16^ approaches (Fig. 2 demonstrates these comparative advantages). This approach eliminates sequential angle-dependent imaging and computational processing requirements, enabling immediate visualization of myelin structure with fiber orientation encoded through color patterns. More details about this approach are in Fig. S1 in the Supplementary Material.

Grayscale imaging employed identical optical configurations with a monochrome camera and a 630±10  nm filter inserted into the filter cube after the light source. qBRM images were acquired using a custom-built microscope described by Gray et al.^16^ A 20× air objective (Olympus, NA = 0.75) provided 0.42-μm lateral resolution, with volumetric imaging using 30-μm z-stacks at 1.3-μm steps, capturing 0.67×0.67-mm field of view per tile.

### 2.4 Annotation Protocol and Dataset Creation

#### 2.4.1 RGB dataset creation and human-in-the-loop training

Using a custom MATLAB annotation GUI, a primary human annotator identified as many visible defects as possible across RGB CCP-BRM volumetric images, followed by a secondary annotator review, retaining only clearly identifiable defects. Annotations were categorized into two classes: general myelin “defects” (including breaks, delaminations, blebbings, swellings, tears, and irregularities) and spherical “lipid vesicles” formed from myelin debris. These defect categories and tissue types were established in our previous comprehensive characterization study,^9^ ensuring consistency with established pathological criteria across control, AD, and CTE conditions. Such defects can impair saltatory conduction of action potentials and contribute to functional decline in neurodegenerative conditions.^3^ Expert consensus annotations were also established following protocols from our previous validation study,^9^ which included expert neuropathologists as co-authors, and where three trained annotators achieved good inter-rater reliability through standardized defect identification criteria.

We first annotated an initial subset of the dataset to bootstrap model training, after which we employed an iterative pseudo-labeling workflow similar to that of Zhang et al.^29^ Pseudo-labeling is a technique in which model predictions on unlabeled samples, referred to as pseudo-labels, are used as labeled data for further training.^30^ In our workflow, each iteration consisted of generating model predictions (i.e., pseudo-labels) on unlabeled images, having annotators review the pseudo-labels to produce expert-verified labels, and retraining the model, incorporating the newly verified labels into the training dataset.

During pseudo-label review, annotators retained correct defect predictions and explicitly marked challenging false positives, which were introduced in the next training iteration as background images. As the review phase did not involve adding new annotations beyond the model’s predictions, we empirically lowered the confidence threshold for generating pseudo labels to minimize the occurrence of false negatives, which would otherwise go uncorrected. Unlike conventional pseudo-labeling,^31^ our human-in-the-loop workflow ensures that only correct model predictions are added to the training set and that the model learns from its false positives, providing richer and more informative feedback in each iteration.

We repeated this workflow for two iterations, after which the model achieved acceptable validation performance (Sec. 3.1). The complete RGB dataset consisted of 3600 defect annotations, 1000 vesicle annotations, and 1000 background annotations. For validation, we reserved an entire RGB image containing 234 defects, 21 vesicles, and 25 background regions.

#### 2.4.2 RGB-to-grayscale dataset conversion for validation

To validate model performance against our rigorously established expert consensus dataset from previous work,^9^ we converted the final RGB training dataset to grayscale format and trained a new model on the resulting grayscale dataset. This conversion utilized green channel replication (GGG format), where the green channel (G) values are duplicated to create red and blue channels, effectively creating a grayscale image while maintaining the three-channel structure required by the model. This approach preserves myelin structural information while ensuring compatibility with our existing grayscale validation dataset.

### 2.5 Data Preprocessing and Augmentation

Raw volumetric stacks (2960×2960  pixels for each of 25 z-planes at 1.3-μm increments) were preprocessed by extracting 100×100  pixel windows around each annotated defect in each z-plane containing the defect, which were then upsampled to 640×640  pixels to match the models’ pretraining resolution. Upsampling the images also enlarged the apparent size of the defects, helping to mitigate challenges associated with small object detection.^32^ To increase the size and diversity of our dataset, we applied standard data augmentation techniques commonly used in prior studies.^33^^,^^34^ These included lighting transformations that modified the hue, saturation, and brightness of the images, and spatial transformations, which involved translation, scaling, and flipping. In addition, we employed the mosaic augmentation,^35^ which stitches together multiple images to simulate complex and densely annotated defect regions.

### 2.6 Model Architecture and Training

We evaluated two state-of-the-art object detection architectures: YOLOv8 Nano^36^ (3.2M parameters) and real-time detection transformer (RT-DETR) large^37^ (42M parameters), both initialized with pretrained weights from the Microsoft Common Objects in Context (MS COCO) dataset. We initially evaluated both architectures but selected YOLOv8 Nano based on its superior computational efficiency and performance on our task (Sec. 3.1).

All experiments were conducted using the Ultralytics training framework^36^ (version 8.3.98) on a single NVIDIA L40 GPU with 48 GB memory for YOLOv8, whereas RT-DETR required three such GPUs due to its significantly larger computational demands. Both models were trained using the AdamW optimizer for 60 epochs, with a batch size of 16. All other training settings were set to default values in the Ultralytics library. We trained and evaluated 100 hyperparameter configurations for each model using the Ultralytics implementation of the genetic algorithm from Bochkovskiy et al.^35^ to determine the optimal learning rate, training schedule, and data augmentation parameters. The best model was selected using mean average precision at 0.5 IoU threshold (mAP@50), as defined by Padilla et al.^38^ To account for the class imbalance between defects and vesicles, we weighted average precision by class frequency when computing this metric. The complete training configuration and optimal hyperparameter values are available in the provided code repository.

### 2.7 Domain Adaptation: Addressing Challenges in Grayscale Validation

The model trained on RGB data converted to grayscale format (Sec. 2.4.2) encountered an unexpected domain shift when applied to authentic grayscale CCP-BRM images from our previous study,^9^ which resulted in increased false positive rates due to transverse axons and blood vessels being misclassified as vesicles. We addressed this by performing three additional iterations of our training workflow with additional brain tissue images acquired in authentic grayscale CCP-BRM format for this domain adaptation purpose: (1) generating predictions on unlabeled images, (2) having experts identify correct predictions and label false positives, and (3) retraining the model with the newly added expert-verified data. To validate model development during this process, we reserved an authentic grayscale CCP-BRM image containing 82 myelin defects and four lipid vesicles. This systematic approach successfully addressed the domain shift between the original green channel replication dataset and authentic grayscale imaging.

### 2.8 Inference and Post-processing Pipeline

Model inference employed a sliding window approach with a 100×100  pixel window, which was upsampled to 640×640  pixels before being input to the model. Consistent with prior studies,^39^ we found that model predictions exhibited sensitivity to small translations in the input image. To address this, we applied 50% overlap between adjacent sliding windows in the vertical and horizontal directions, followed by nonmaximum suppression^40^ to eliminate redundant predictions due to the overlap. Postprocessing also performed z-plane filtering, which automatically removed vesicle detections that appeared in three or more consecutive z-planes, as these are likely false positives, typically transverse axons [see Fig. 1(c), right-hand panel].

For large-scale analysis, defect density heatmaps were generated by counting detected defects within each sliding window and applying a color overlay to visualize the spatial distribution of pathology.

### 2.9 Evaluation Methodology

Model performance was evaluated using standard object detection metrics, including mAP@50, F1 score, precision (positive predictive value), and recall (sensitivity).^38^ These metrics were calculated as: precision = TP / (TP + FP), recall = TP / (TP + FN), F1 score = 2 × (precision × recall) / (precision + recall), where TP, FP, and FN represent true positives, false positives, and false negatives, respectively. mAP@50 was calculated according to the definition of Padilla et al.^38^

Model predictions were also validated against an independent dataset of 200 full images (600×600  pixels for each of 23 z-planes at 1.3-μm increments) from our previous paper,^9^ processed using the 100×100 sliding window approach described in Sec. 2.8. Defects were considered true positives if identified by at least two of three expert annotators, with minimum 20% IoU overlap and ±1 z-plane tolerance.

Linear correlation analysis assessed relationships between human and automated annotation counts across disease conditions using statistical software IBM SPSS Statistics (version 27). Inter-rater reliability was evaluated using two complementary analyses: (1) SPSS reliability analysis with a two-way mixed effects model for single measures, calculating intraclass correlation coefficients (ICCs) with 95% confidence intervals;^41^ and (2) bivariate correlation analysis (Pearson correlations) to determine statistical significance of individual pairwise relationships between all raters.^42^ Statistical significance was set at α=0.05. Given the six pairwise comparisons among four raters, Bonferroni correction was applied to control for multiple comparisons (corrected α=0.0083). Both analyses used two-tailed significance testing.

## 3 Results

**Fig. 3:**
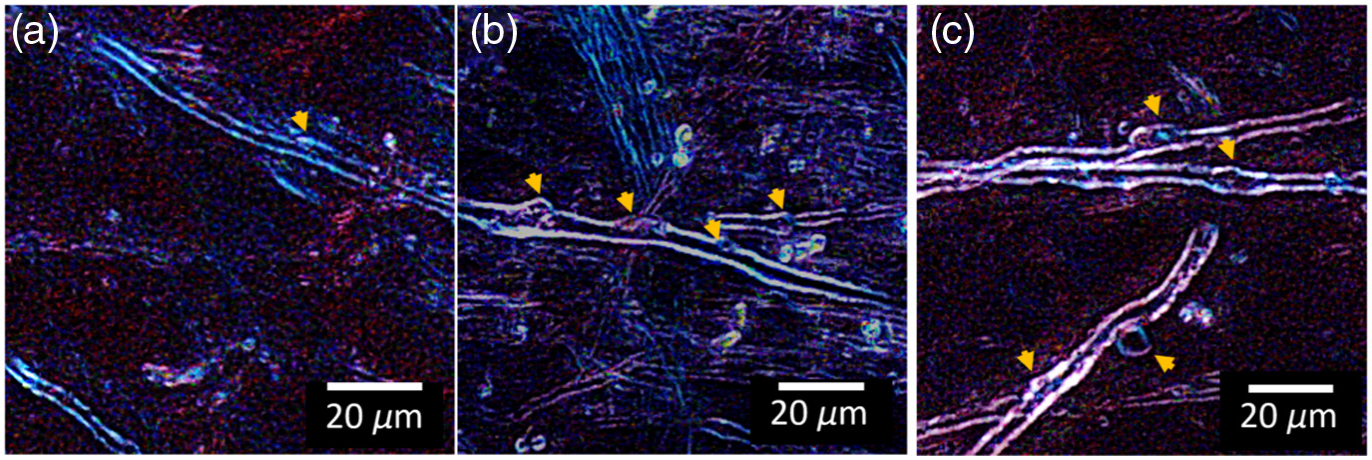
Automated detection performance across representative tissue types from each disease condition. Panel (a) shows control tissue with normal myelin architecture, panel (b) shows AD tissue, and panel (c) shows CTE tissue. Yellow arrows indicate detected defects in each case. This demonstrates the model’s ability to detect pathological features across the spectrum of tissue morphologies encountered in neurodegenerative conditions.

Our results demonstrate successful extension of automated myelin defect detection of human cortical gray matter affected by neurodegenerative pathology in birefringence microscopy images, representing the validation of computational object-detection approaches in this complex tissue environment. Figure 3 demonstrates automated detection examples across representative tissue types from each disease condition.

### 3.1 Model Performance and Detection Accuracy

**Fig. 4:**
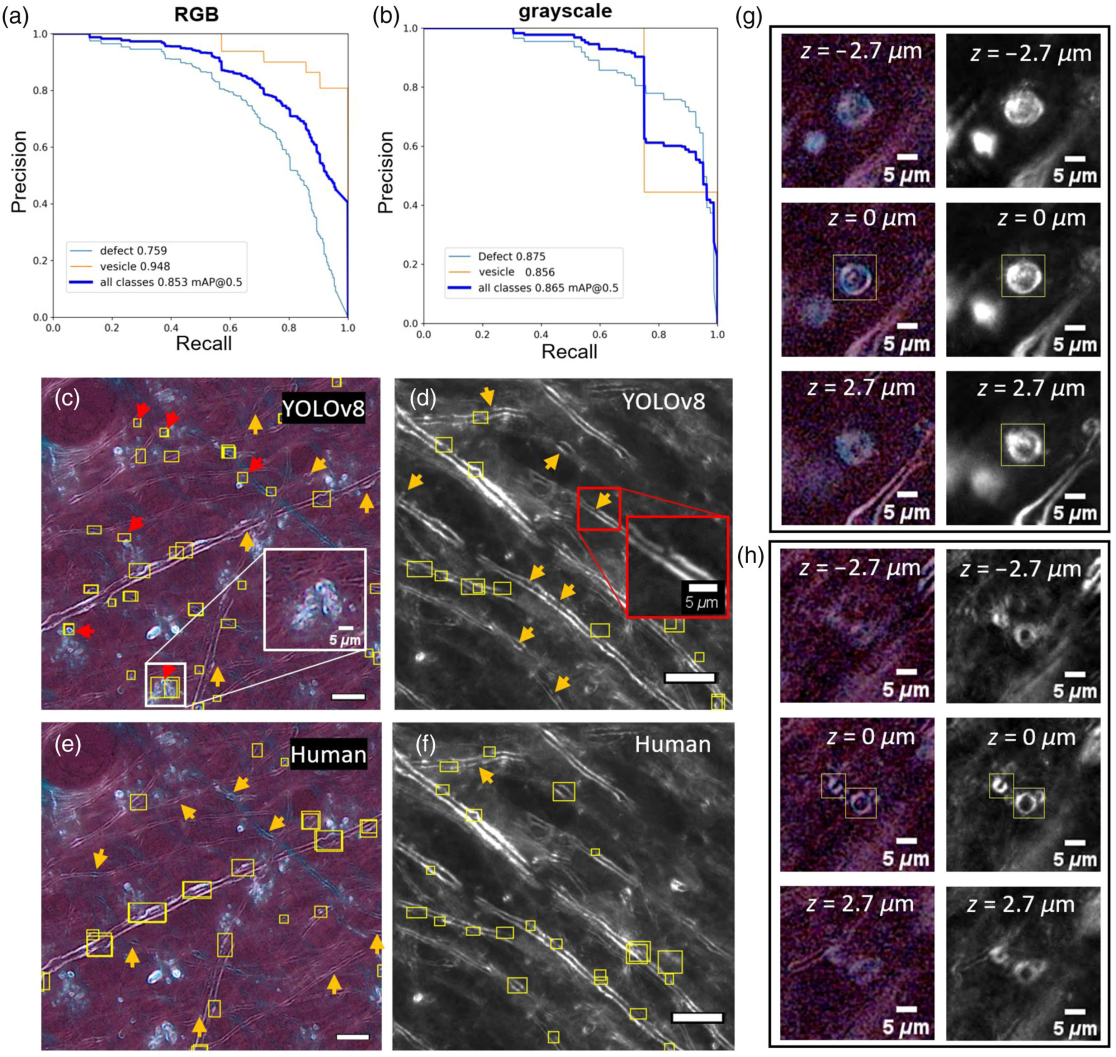
YOLOv8 model performance demonstrates high accuracy for automated myelin defect detection across imaging modalities. Precision-recall curves showing strong performance for both (a) the RGB model and (b) the grayscale model across defect classes. (c), (d) Representative automated detections (yellow bounding boxes) compared to (e), (f) expert manual annotations, where red arrows point to false positive detections (mostly transverse axons) and orange arrows point to missed defects by the model/human. Representative examples shown; arrows highlight specific cases for illustration and do not represent overall error rates across the validation dataset. Zoom-in insert in (c) shows a false positive defect, that is, a bundle of transverse axons, and (d) shows a small false negative defect missed by the model. (g), (h) Representative examples of vesicle detection across consecutive z-planes in RGB CCP-BRM (left) and grayscale CCP-BRM (right) from CTE patient tissue. (c), (e) are focus-stacked RGB CCP-BRM images from AD patient tissue, (d), (f) are grayscale focus-stacked CCP-BRM images from CTE patient tissue. Scale bars in (c)–(f): 20  μm.

YOLOv8 Nano demonstrated an optimal performance-efficiency balance with mAP@50 of 0.85 and F1 score of 0.77 for RGB imaging, compared with RT-DETR large at 0.82 mAP@50, while requiring significantly fewer computational resources (3.2M versus 42M parameters). Performance varied by class: for myelin defects, AP@50 of 0.75 was achieved, whereas AP@50 of 0.94 [Fig. 4(a)] was achieved for vesicles.

Following domain adaptation, the grayscale model achieved mAP@50 of 0.865 (F1=0.82) [Fig. 4(b)], demonstrating successful transfer to authentic imaging conditions with improved precision (0.9 versus 0.75), reflecting targeted false-positive reduction, whereas the application of the RGB CCP model maintained higher recall (0.81 versus 0.75) for extensive defect detection.

Figure 4 demonstrates representative model predictions on single validation images selected to illustrate different detection outcomes. These examples should not be interpreted as representative of performance across our complete validation dataset. The mAP@50 metric integrates performance across all confidence thresholds, so overall performance may appear inconsistent with individual illustrative examples.

Comparative analysis reveals distinct error patterns between the two models: the RGB model [Fig. 4(c)] exhibits higher false positive rates but fewer missed detections, whereas the grayscale model [Fig. 4(d)] shows reduced false positives at the cost of increased false negatives. This trade-off directly corresponds to the observed precision-recall differences, with the grayscale model’s enhanced precision reflecting successful false-positive reduction through iterative training, whereas the RGB model’s higher recall indicates superior sensitivity for defect detection.

The RGB model also exhibits higher vesicle detection accuracy (AP@50 = 0.94 versus 0.86 for grayscale), though the statistical significance of this difference is uncertain given the limited size of the dataset. Analysis of vesicle appearance reveals distinct characteristics across imaging modalities [Figs. 4(g) and 4(h)]. In RGB CCP-BRM, vesicles exhibit sharper features at optimal focus (z=0  μm) and rapid signal reduction at adjacent z-planes (z=±2.7  μm). By contrast, grayscale CCP-BRM shows vesicles maintaining visibility across a broader range of z-planes. The color information in RGB images provides enhanced visual contrast between vesicles and morphologically similar structures such as transverse axons.

### 3.2 Validation across Disease Conditions

**Fig. 5:**
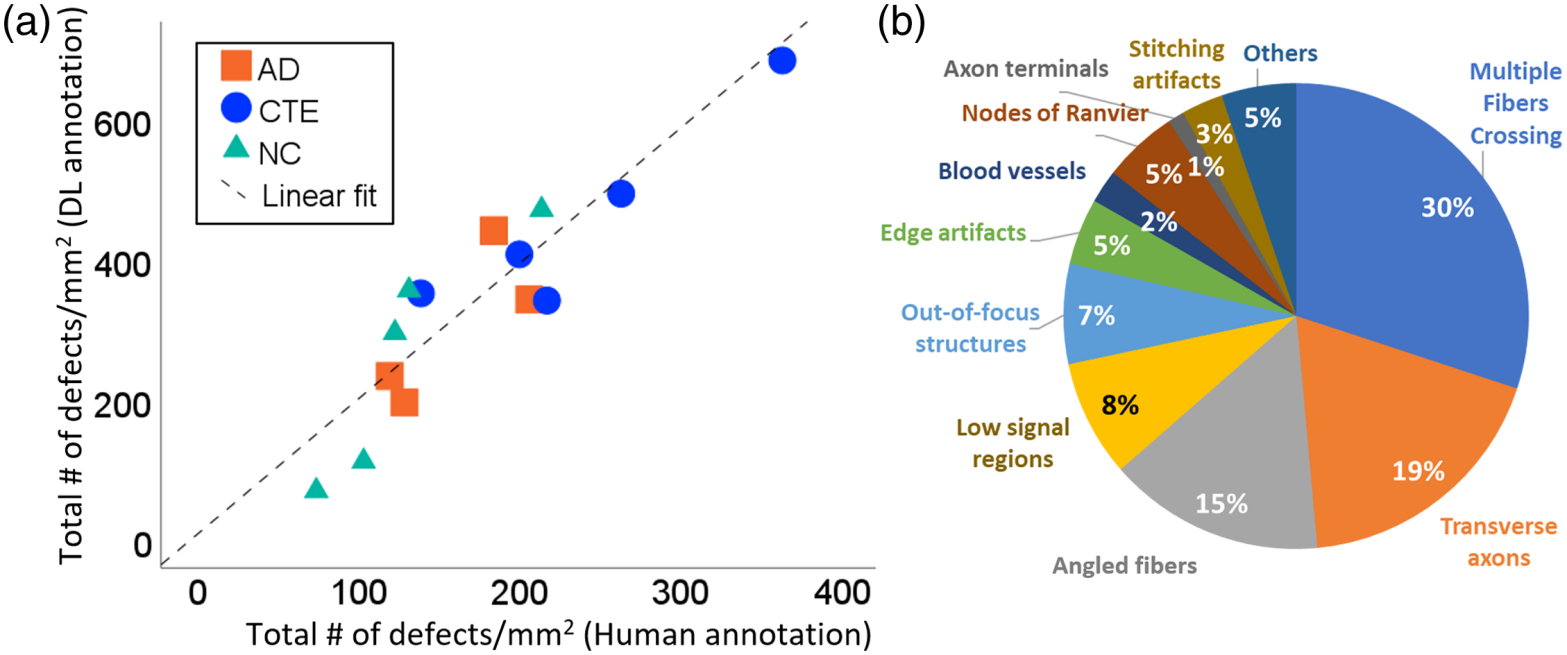
Cross-disease validation of automated detection. (a) Strong linear correlation (r=0.902, p<0.001, $R^{2}=0.81$) between automated predictions and expert consensus across neurodegenerative conditions. Controls (triangles, n=5), Alzheimer’s disease (squares, n=5), chronic traumatic encephalopathy (circles, n=5). The preserved linear relationship validates automated detection for comparative disease studies. The dashed line indicates a linear fit. (b) Breakdown of false positive categories from systematic analysis of 173 false positives identified in 100 validation images.

Cross-disease validation confirmed the reliability of automated detection across pathological conditions. Critically, the linear relationship between human and automated counts (Pearson r=0.902, p<0.001, $R^{2}=0.81$) was preserved across AD, CTE, and normal control samples [Fig. 5(a)], indicating that relative disease patterns remain consistent between human and automated approaches despite domain adaptation challenges.

Automated detection consistently identified more defects per image than expert consensus [Fig. 5(a)]. This reflects our conservative validation methodology requiring 2-of-3 annotator agreement, which inherently excludes some genuine defects identified by only one expert. Visual verification of additional detections confirmed that these mostly represent genuine myelin pathology with 11.3% false positives. More detailed FP analysis is provided below. The preserved linear relationship validates comparative disease studies despite absolute count differences.

Analysis of 100 validation images revealed specific performance characteristics and remaining challenges. From 1529 total detections, 173 were false positives (11.3% false positive rate, 88.7% precision on this subset). The most common false positives [Fig. 5(b)] were due to fiber morphology variations: multiple fibers crossing (30.1%), transverse axons appearing as circular structures similar to vesicles (18.5%), and angled fibers with ambiguous defect-like features (15.0%). Image quality issues represented the second major category, including low signal regions (8.1%), out-of-focus myelin structures (6.9%), and edge artifacts (4.6%).

Importantly, blood vessels with vesicle-like morphology represented only 2.3% of false positives, indicating that our domain adaptation successfully addressed this initially prominent challenge through iterative training. Normal anatomical structures occasionally triggered false positives, including nodes of Ranvier (5.2%) and axon terminals (1.2%). Technical artifacts from image stitching contributed 2.9% of false positives.

### 3.3 Inter-rater Reliability

Inter-rater reliability analysis revealed good agreement among all four raters (three expert annotators plus the deep learning model). The ICC was 0.686 (95% CI: 0.613 to 0.748), indicating good reliability.^43^

Individual correlations between the deep learning model and expert annotators (Table 2) ranged from 0.491 to 0.611 (all p<0.001, significant after Bonferroni correction for multiple comparisons), demonstrating that automated performance approaches human expert reliability standards.

**Table 2:** Inter-rater reliability analysis results from SPSS reliability analysis.

Rater pair	Pearson correlation	Significance
Expert 1 versus expert 2	0.352	p<0.001
Expert 1 versus expert 3	0.238	p<0.001
Expert 2 versus expert 3	0.509	p<0.001
Deep learning versus expert 1	0.491	p<0.001
Deep learning versus expert 2	0.611	p<0.001
Deep learning versus expert 3	0.501	p<0.001

Item statistics represent the mean (μ) and standard deviation (σ) of defect counts per image for each rater, showing the detection patterns across all validation images: expert 1 (μ=13.42, σ=10.17), expert 2 (μ=6.12, σ=4.27), expert 3 (μ=5.37, σ=3.31), deep learning model (μ=13.38, σ=9.86).

### 3.4 Processing Efficiency and Workflow Analysis

The human-in-the-loop automated-detection approach achieved a 14-fold time reduction compared with pure human annotation, from 8 h to 33 min per $1\;{\mathrm{mm}}^{2}$ area of tissue section (3 min automated inference +30  min expert verification). Large-scale processing of 300 GB volumetric images required ∼32 GPU hours for model inference, enabling large-volume ($∼15\;mm^{3}$) analysis with comprehensive spatial mapping of myelin pathology distribution.

**Fig. 6:**
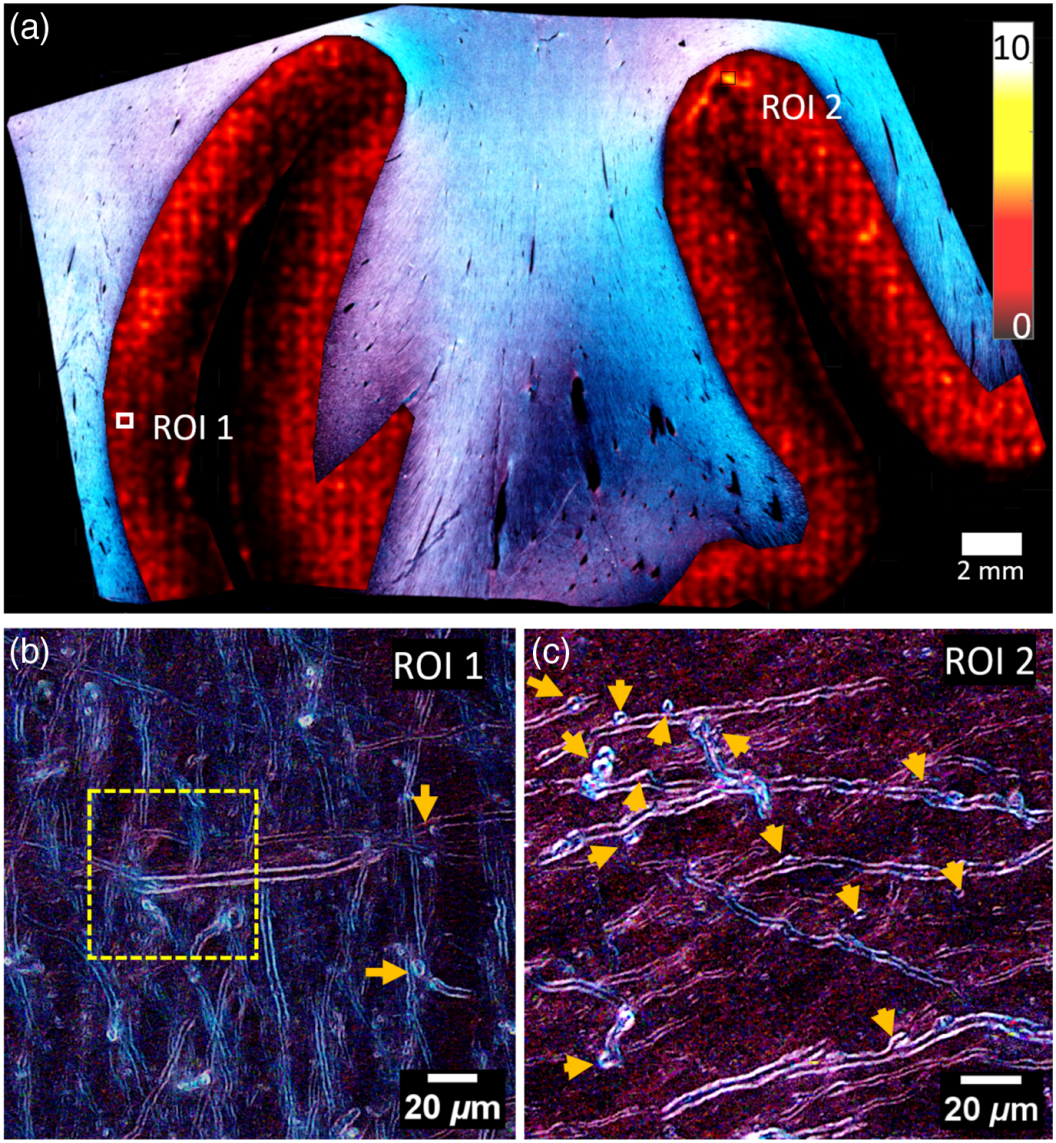
Large-scale automated tissue analysis capabilities. (a) Representative CTE tissue section with automated defect density heatmap overlay (dark = low density, bright = high density). The heatmap colors in GM represent quantified defect counts per sliding window, while in WM they represent fiber orientations. (b) Low-defect density region showing normal myelin architecture (dashed yellow square indicates sliding window size used for heatmap generation). (c) High-defect density region with numerous pathological features (arrows indicate myelin defects). (a)–(c) are focus-stacked RGB CCP-BRM images.

Figure 6 demonstrates large-scale tissue analysis capabilities with automated defect-density mapping. The color overlay represents defect counts per sliding window analysis, providing spatial visualization of pathology distribution across the tissue section. The heatmap overlaid on a representative RGB CCP-BRM image of a tissue section from a CTE subject, showing both low-defect density regions with normal myelin architecture [Fig. 6(b)] and high-defect density areas with numerous pathological features [Fig. 6(c)]. The same density mapping approach can be applied to the grayscale CCP-BRM image (see Fig. S2 in the Supplemental Material).

## 4 Discussion

Our results show that human-in-the-loop deep learning achieves precision and recall in detecting myelin defects comparable to values reported in previous work^44^^,^^45^ while providing dramatic efficiency improvements essential for large-scale pathological studies. The strong correlation between automated detections and expert consensus across different pathological conditions validates this approach for comparative disease studies, addressing one of the bottlenecks in neurodegeneration research, where myelin dysfunction is increasingly recognized as playing an important role in disease pathogenesis.^5^^–^^8^

### 4.1 Technical Innovations and Implementation Advantages

This work represents an application of RGB CCP-BRM imaging for automated pathological analysis in human neurodegenerative tissue, addressing both acquisition-time and processing bottlenecks simultaneously. The RGB CCP-BRM approach addresses a critical bottleneck in creating high-quality training datasets for automated detection. Although automated model performance between RGB and grayscale approaches is comparable (0.85 versus 0.865 mAP@50), the primary advantage lies in enhanced defect visibility for human annotators during initial dataset creation.

Our RGB CCP-BRM implementation offers several critical advantages over traditional qBRM approaches for dataset development and annotation workflows. Unlike qBRM, which requires sequential angle-dependent imaging and computational post-processing, RGB CCP-BRM provides real-time fiber orientation visualization with at least 3× faster acquisition than qBRM. Most importantly for training dataset quality, the color-encoded orientation information makes structural disruptions significantly more apparent to human annotators, enabling more complete and accurate manual annotations that serve as ground truth for model training. The wavelength-dependent polarization effects create distinct visual signatures that highlight myelin irregularities, breaks, and delaminations that appear less conspicuous in traditional grayscale approaches, directly improving annotation quality and consistency. In addition, the technique’s compatibility with standard microscopy equipment significantly lowers adoption barriers compared to specialized quantitative birefringence systems.

The reasons for the observed difference in vesicle detection performance between RGB and grayscale imaging are not yet established. The limited size of our dataset prevents a definitive assessment of whether this difference is statistically significant. We can only speculate about potential mechanisms if the performance difference is real. Chromatic aberration offers a plausible explanation for the vesicle appearance difference between modalities: the broader wavelength range in RGB CCP-BRM experiences wavelength-dependent focusing, where different wavelengths focus at slightly different z-positions, potentially creating a blurrier appearance in out-of-focus planes. In addition, color information may provide discriminative features for distinguishing vesicles from morphologically similar structures. However, these remain hypotheses requiring controlled validation experiments with larger, matched datasets to establish both statistical significance and underlying mechanisms.

Our approach achieved 0.85 mAP@50, comparing favorably with other YOLO-based pathology applications^20^^,^^46^ while maintaining practical deployment feasibility for research laboratories. The automated performance approaches human expert reliability standards, with inter-rater correlations ranging from 0.491 to 0.611, validating its use for comparative research studies.

Domain adaptation proved critical, revealing that substantial performance shifts can occur with imaging parameter changes, but systematic iterative training successfully addressed these challenges.^47^

### 4.2 Research Applications

The 14-fold time reduction from 8 h to 33 min per ${\mathrm{mm}}^{2}$ enables comprehensive assessment across entire tissue sections rather than limited regions, significantly improving statistical power for mechanistic studies of myelin pathology.^48^ This efficiency gain makes previously impractical large-scale investigations feasible, potentially revealing insights into disease mechanisms.^49^

Beyond post-mortem analysis, RGB CCP-BRM’s real-time acquisition enables broader research applications, including *ex vivo* organotypic cultures, animal models, and therapeutic screening studies.^50^^–^^52^ This approach supports large-scale screening of potential therapeutics, assessment of treatment efficacy, and longitudinal studies of myelin dynamics that complement post-mortem analysis.^53^

Current diagnostics approaches rely on a combination of conventional histological staining of post-mortem tissue and MRI-based diffusion tensor imaging in live patients,^54^^,^^55^ which operate at different scales and sensitivity levels compared with microscopic birefringence analysis.^56^ Our approach provides complementary *ex vivo* assessment that could inform interpretation of *in vivo* MRI findings and support mechanistic understanding for therapeutic development.^57^

For clinical translation, this approach serves as a research tool for advancing fundamental understanding of myelin pathology mechanisms. The quantitative analysis provides mechanistic insights that can guide therapeutic development, supports correlation between microscopic pathology and clinical imaging biomarkers, and enables more consistent neuropathological assessment crucial for clinical trial endpoints. The primary value lies in developing research frameworks that may ultimately inform therapeutic strategies targeting myelin preservation.^58^

### 4.3 Limitations and Technical Constraints

Several important limitations constrain the current implementation. The method is optimized for fibers parallel to the imaging plane, with reduced sensitivity for highly angled or perpendicular fibers. This constraint limits comprehensive analysis of regions with complex three-dimensional fiber architectures, though it does not affect the detection of pathological features in the predominant parallel fiber populations studied here. Current clinical standards accommodate this limitation using multiple complementary approaches (e.g., sections cut at different orientations, volumetric MRI) to provide a more comprehensive assessment.

Systematic analysis of failure modes reveals insights into the fundamental challenges of myelin pathology detection. Complex fiber crossing patterns (30.1% of false positives) reflect the inherent difficulty of distinguishing pathological changes from normal anatomical complexity. Transverse axon misclassification (18.5% of false positives) and angled fibers with ambiguous features (15.0%) represent additional challenges that may require specialized algorithms for anatomical structure recognition.

The classification strategy we employed grouped 78.3% of annotations into a general myelin “defect” class, which simplified dataset creation and model training but limited analyses requiring distinction among specific defect morphologies. In addition, RGB CCP-BRM provides qualitative fiber orientation visualization rather than the quantitative birefringence measurements available with the previously established qBRM approaches.^14^^,^^16^ Although this trade-off enables faster acquisition and real-time assessment, it limits the quantitative characterization of myelin structural properties.

The current validation focuses on post-mortem human cortical tissue from specific brain regions (dorsolateral prefrontal cortex) and pathological conditions (AD, CTE, controls). Clinical translation would require extensive validation across different tissue types, disease stages, brain regions, and potentially adaptation for biopsy-based analysis. Although the methodology shows promise for research applications, direct clinical diagnostic use would necessitate rigorous validation studies comparing automated detection results with established clinical standards and patient outcomes.

### 4.4 Future Directions

Future development should focus on addressing complex fiber morphology patterns through expanded training datasets and specialized algorithms for anatomical structure recognition to reduce false positive rates. The predominance of fiber crossing patterns among false positives suggests that incorporating additional contextual information, such as local fiber orientation patterns, could improve discrimination between pathological changes and normal anatomical complexity.

Adopting a classification strategy that captures specific defect subtypes can also extend our approach for fine-grained pathological investigations. Integration with other microscopy techniques could enable previously impractical large-scale studies across neurodegenerative disorders and provide additional information about myelin structure and composition.^49^^,^^59^

As model robustness improves through expanded training datasets, future implementations may achieve purely automated detection for routine applications, eliminating the verification step and potentially achieving greater than 14-fold time improvements. However, expert oversight will likely remain essential for research applications requiring high accuracy and for handling novel pathological patterns not represented in training data.

Technical improvements could include utilizing multi-order quarter-waveplates in the circular polarizers, in lieu of the zero-order waveplates used for the reported results. This would result in larger dispersion values of the birefringence for illumination and detection, thus generating larger color changes as a function of fiber orientation, and consequently improved SNR.

## 5 Conclusion

We developed and validated a human-in-the-loop deep learning approach combining RGB CCP-BRM imaging with automated defect detection for myelin pathology analysis in complex human cortical tissue. This approach reduces analysis time 14-fold while maintaining a strong correlation to expert consensus across neurodegenerative conditions.

The successful domain adaptation and application to complex human pathology demonstrate the practical potential of this technology. This enables comprehensive research investigations of myelin-pathology relationships that were previously impossible due to throughput limitations, potentially advancing our fundamental understanding of myelin dysfunction mechanisms in neurodegenerative disorders.

## Supplementary Material

10.1117/1.NPh.12.4.045007.s01

## Data Availability

The MATLAB annotation GUI along with the code and trained models of our method are available at https://github.com/arjunchandra2/Myelin-Defect-Detection. The dataset is available at https://universe.roboflow.com/defecttraining/myelin-defect-detection-s89w2.
