# Supplementary material for: Accelerating myelin defect detection in neurodegenerative disorders: a human-in-the-loop deep learning approach with birefringence microscopy
Source: Neurophotonics. 2025 Nov 4;12(4):045007. doi: 10.1117/1.NPh.12.4.045007 (PMC12585155; doi:10.1117/1.NPh.12.4.045007)
Supplement: Supplementary file 1 [file NPh_012_045007_SD001.pdf]

Supplementary material A

Methodological Validation Against qBRM

Figure S1 demonstrates methodological validation against established qBRM approaches rather than operational workflow steps. This comparison validates that our RGB CCP-BRM implementation can extract equivalent quantitative information to the established methods, while providing operational advantages of real-time acquisition without sequential imaging or post-processing requirements.

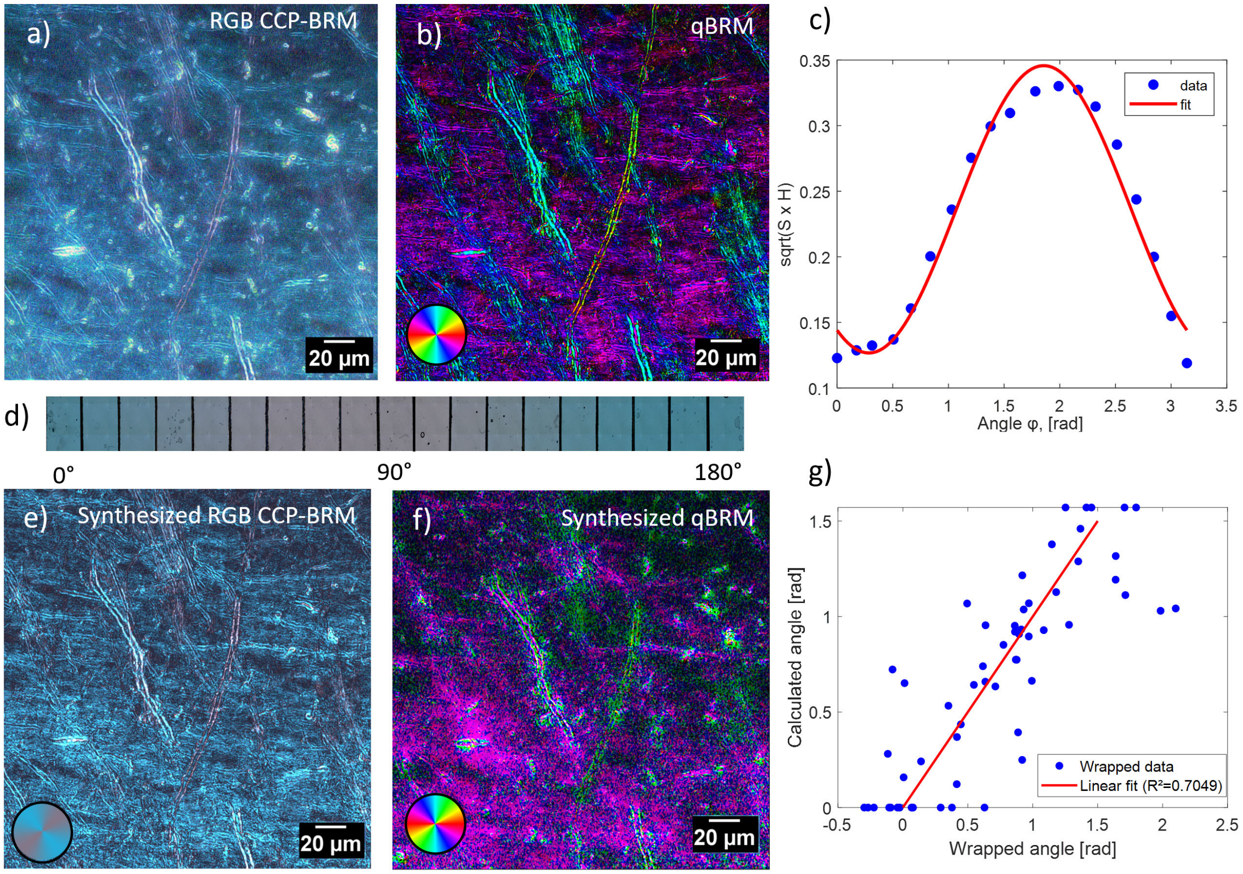

Figure S1. RGB CCP-BRM imaging system calibration and validation against qBRM methods. This comparison validates that RGB CCP-BRM can extract equivalent quantitative information while providing operational advantages of real-time acquisition. (a) Authentic RGB CCP-BRM image of control brain sample. (b) Traditional qBRM image with color wheel indicating in-plane optic axis orientation. (c) Calibration curve showing sinusoidal fit between $y(\varphi) = \sqrt{Saturation \times Hue}$ and known angles from calibration slide (d), sinusoidal fit done using equation S1. (d) Linear polarizer calibration slide image (0°-180°) for color-angle mapping. (e) Synthesized RGB CCP-BRM image generated using custom colormap extracted from calibration slide image. (f) Synthesized qBRM image from RGB CCP-BRM showing fiber orientation-dependent color encoding. (g) Angle correlation validation plot between qBRM measured angles and angles extracted from RGB CCP-BRM image using equation S2 ($R^2 = 0.7049$). (a, b, e, f) are focus-stacked images. Color wheel in (a, b, c) indicates in-plane optic axis orientation. Scale bars: 20 μm.

Calibration and Quantitative Analysis

To enable quantitative analysis and demonstrate results equivalent to established qBRM, we developed a mathematical conversion method from RGB CCP-BRM to equivalent qBRM

measurements. RGB images were converted to HSB (Hue-Saturation-Brightness) color space for analysis.

The calibration process involved imaging a linear polarizer array at known orientations from 0° to 180° in 10° increments (Figure S1d). For each angular position, we extracted the color parameter y(φ) = √(Saturation × Hue) from small ROIs (5×5 pixels) and fitted these values to the sinusoidal relationship:

$$y(\varphi) = A \cdot \sin(2 \cdot \varphi + B) + C, \qquad (S1)$$

where $y(\varphi)$ represents the measured color parameter and φ is the orientation angle. The fitting procedure yielded calibration parameters A, B, and C through least-squares optimization of Equation S1 to the measured calibration data. While A represents the amplitude of the sinusoidal relationship, B represents a phase shift, and C represents the baseline offset, the physical interpretation of the parameters is not definitively established from our calibration procedure. Multiple color parameter combinations were tested, with $y(\varphi) = \sqrt{Saturation \times Hue}$ providing optimal correlation ($R^2 = 0.97$ with calibration slide).

The angle extraction equation was derived through standard trigonometric inverse operations:

$$\varphi(y) = \left(-\frac{1}{2}\right) \times \sin^{-1}\left(\frac{y - C}{A}\right) + \frac{B}{2}, \qquad (S2)$$

Validation with Tissue Images

To validate calibration accuracy, we applied Equation S2 to extract angles from coregistered RGB CCP-BRM images and compared these with angles measured from corresponding qBRM images of the same tissue regions, achieving $R^2 = 0.70$ correlation (Figure S1g). This validation confirms that the calibration relationship accurately converts RGB CCP-BRM color information to quantitative orientation measurements comparable to established qBRM methods.

Figure S1g demonstrates the relationship between angles extracted from Equation S2 and measured angle values wrapped around 90°, confirming the linear relationship between the two modalities. This 90° periodicity occurs because the same y(φ) value corresponds to angles 90° apart (e.g., 1 rad and 2.57 rad in Figure S1c). Due to original RGB CCP-BRM image noise, the synthesized qBRM image lacks details present in the original qBRM image, and noise is reflected in Figure S1g as deviations from the linear relationship.

The 90° periodicity in fiber orientation measurements is inherent to birefringence imaging, where the same birefringence signal corresponds to angles 90° apart. This fundamental limitation constrains complete angular characterization. However, this periodicity does not affect defect detection accuracy, which relies on structural disruption identification rather than precise orientation measurements.

Synthesized Image Generation

Synthesized RGB CCP-BRM images (Figure S1e) were generated by applying a custom colormap extracted from calibration slide image (Figure S1d), where each angle was assigned a custom color instead of traditional rainbow pattern. Synthesized qBRM images (Figure S1f) were generated by multiplying the angle map by brightness, where the angle map was extracted using Equation S2, and brightness was obtained from the HSB transformation.

The calibration methodology (Figure S1e-f) validates that RGB CCP-BRM can extract equivalent quantitative information to established qBRM approaches, providing scientific confidence in our approach while maintaining the operational advantages of real-time acquisition.

Supplementary material B

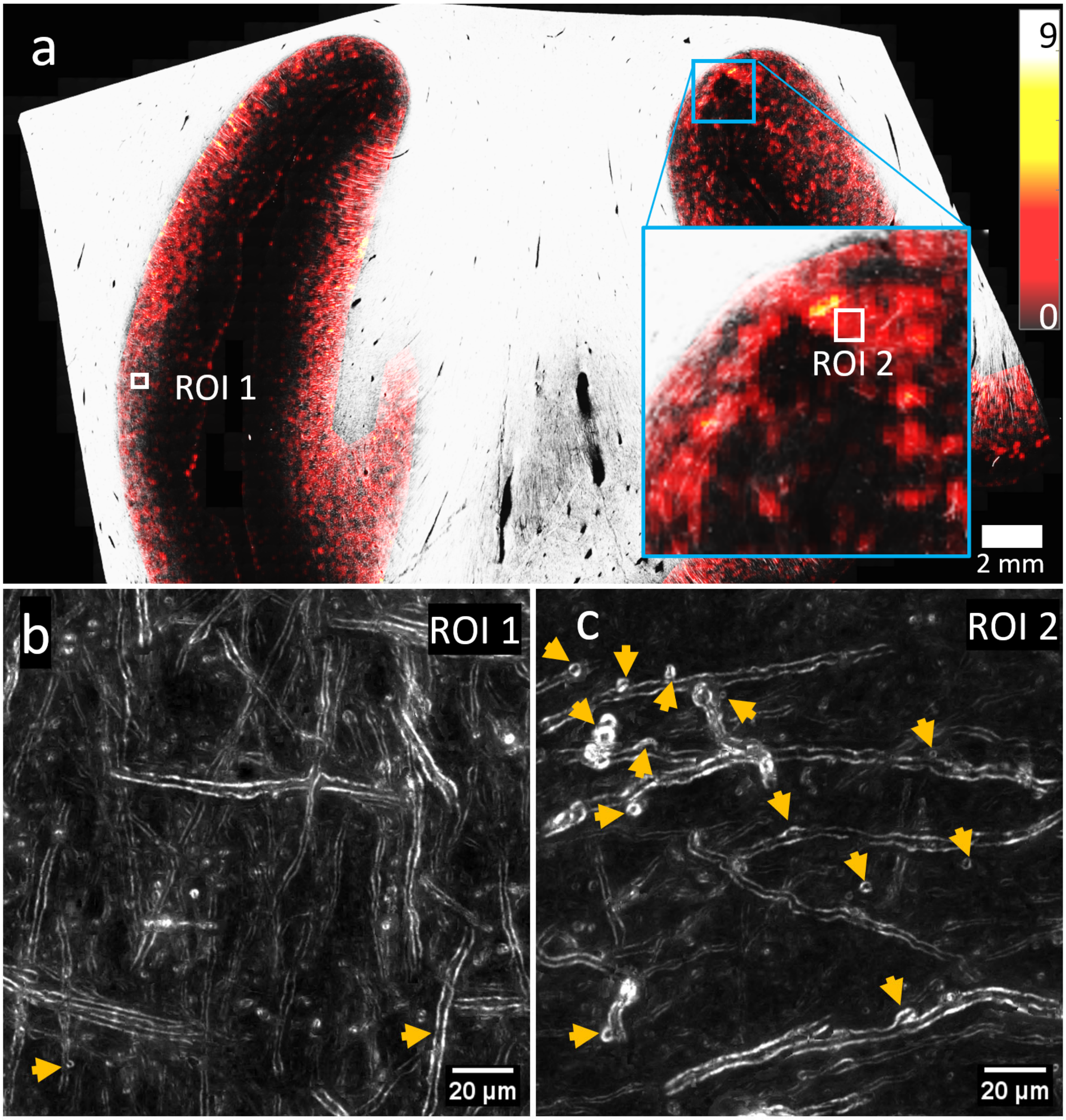

Figure S2. Large-scale automated tissue analysis capabilities. (a) Representative CTE tissue section with automated defect density heatmap overlay (dark = low density, bright= high density). The heatmap colors represent quantified defect counts per sliding window, not fiber orientations (b) Low-defect density region showing normal myelin architecture. (c) High-defect density region with numerous pathological features (arrows indicate myelin defects). (a, b, c) are focus-stacked grayscale CCP-BRM images.
